# Left Renal Vein Pseudo-Fenestration

**DOI:** 10.3390/reports9030299

**Published:** 2026-09-06

**Authors:** Daniel Gondorf, George Triantafyllou, Nikolaos-Achilleas Arkoudis, Georgios Velonakis, Kostis Gyftopoulos, Maria Piagkou

**Affiliations:** 1 Department of Anatomy, School of Medicine, Faculty of Health Sciences, National and Kapodistrian University of Athens, 11527 Athens, Greece; daniel.gondorf@gmail.com (D.G.); mapian@med.uoa.gr (M.P.); 2 Research Unit of Radiology and Medical Imaging, National and Kapodistrian University of Athens, 11527 Athens, Greece; nick.arkoudis@gmail.com (N.-A.A.); gvelonakis@med.uoa.gr (G.V.); 3 Second Department of Radiology, General University Hospital “Attikon”, National and Kapodistrian University of Athens, 12462 Athens, Greece; 4 Department of Anatomy, School of Medicine, Faculty of Health Sciences, University of Patras, 26504 Rion, Greece; kogyftop@upatras.gr

**Keywords:** left renal vein, pseudo-fenestration, vascular variation, multidetector computed tomography

## Abstract

We describe a rare case of LRV pseudo-fenestration in a 68-year-old female identified on contrast-enhanced multidetector computed tomography. Unlike true fenestration, in which a single vessel splits and reunites, this pseudo-fenestration resulted from the convergence of two distinct hilar venous trunks. Multiplanar and three-dimensional reconstructions demonstrated that the left suprarenal vein drained into the superior limb, whereas the left gonadal vein drained into the inferior limb, reflecting a consistent and anatomically meaningful tributary pattern. This configuration is most plausibly explained by incomplete or asymmetric embryological fusion of the cardinal venous system. Accurate preoperative recognition of this variant is clinically important to reduce the risk of iatrogenic injury during laparoscopic donor nephrectomy, adrenal venous sampling, and other retroperitoneal or endovascular procedures.

**Figure 1:**
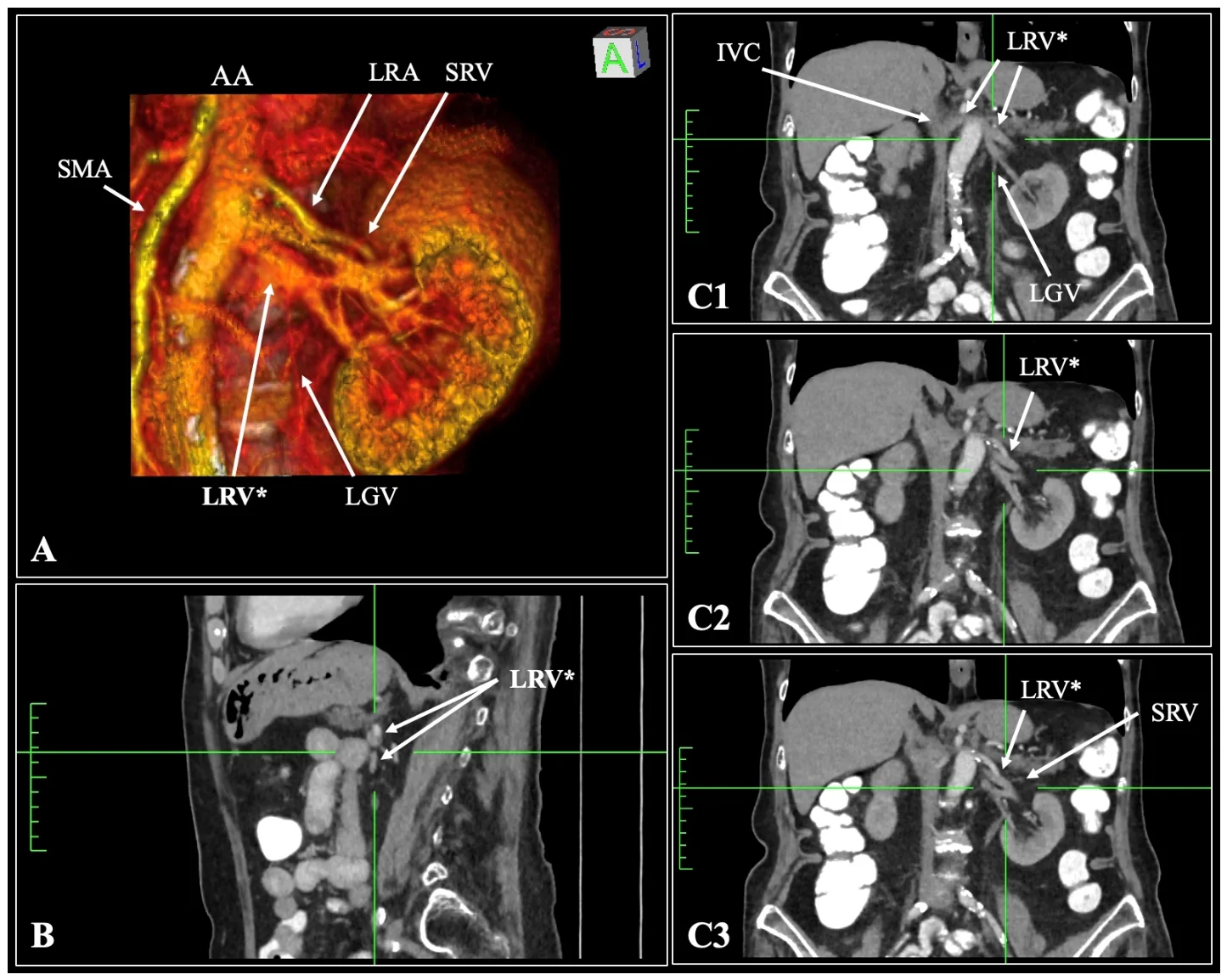
Three-dimensional (**A**) and multiplanar (**B**,**C1**–**C3**) reconstructions of a 68-year-old female patient who underwent contrast-enhanced multidetector computed tomography (CT) of the abdomen are presented, with a slice thickness of 1.0 mm. The examination was performed using a multidetector CT scanner (Canon Medical Systems, Tochigi, Japan). Imaging was acquired using a contrast-enhanced biphasic abdominal CT protocol. Intravenous iodinated contrast medium (370 mg iodine/mL) was administered via a power injector at a rate of approximately 4 mL/s, followed by a saline flush. The datasets were post-processed and analyzed using Horos software version 3.3.6 (Horos Project, Annapolis, MD, USA). Image interpretation was based on multiplanar reconstructions (axial, coronal, and sagittal) and three-dimensional volume-rendered reconstructions, enabling comprehensive evaluation of the renal vascular anatomy. The case was identified during a previous retrospective study [1], where patients were completely anonymized except for their demographic characteristics; thus, the clinical history and the etiology obtained from the CT are not available. The pseudo-fenestrated left renal vein (LRV*) is formed by two hilar veins that exit the kidney hilum. The two limbs (superior and inferior) can be observed in sagittal reconstruction (**B**). The complete course of the LRV* is depicted in three consecutive coronal reconstructions (**C1**–**C3**). The suprarenal vein (SRV) drains into the superior limb, and the left gonadal vein (LGV) drains into the inferior limb. Two distinct venous trunks emerge from the left renal hilum, with the superior vein measuring 5.29 mm in diameter and the inferior vein measuring 4.67 mm. At a distance of 17.14 mm from the renal hilum, the superior venous trunk participated in the formation of a venous window measuring 16.05 mm in length, consistent with a fenestration-like configuration. The inferior hilar vein joined the inferior limb of this configuration at approximately 4.45 mm distal to its origin, resulting in a convergent venous pattern rather than a true dividing channel. The left gonadal vein was observed draining into the inferior limb, whereas the left suprarenal vein drained into the superior limb, demonstrating a reproducible and anatomically consistent tributary pattern. The anatomical configuration was clearly demonstrated on three-dimensional volume-rendered images (**A**) and further validated on multiplanar reconstructions, particularly on sagittal (**B**) and sequential coronal images (**C1**–**C3**), which enabled continuous tracking of the venous course from the renal hilum to the inferior vena cava. Coronal reconstructions provided the most informative depiction of the spatial relationship between the two venous limbs and their point of convergence (**C1**–**C3**), as illustrated in the Supplementary Video. The literature on LRV fenestration remains limited. Gündoğdu et al. [2] provided one of the earliest imaging-based documentations of true LRV fenestration using CT angiography, while subsequent anatomical and cadaveric studies have broadened the recognized morphological spectrum of this entity. Watanabe et al. [3] further demonstrated that renal, gonadal, suprarenal, or ovarian arteries may traverse the fenestrated segment, underscoring the close spatial and developmental relationships between the arterial and venous systems. Damen et al. [4] offered the first structured evaluation of this variation, reporting a prevalence of 3.34%, defining its topographical distribution (preaortic, para-aortic, or distant), and clearly distinguishing true from pseudo-fenestrations. They also described a consistent tributary pattern, with the suprarenal vein draining into the superior limb and the gonadal vein draining into the inferior limb. Crucially, the distinction between true fenestration (division of a single venous trunk) and pseudo-fenestration (convergence of two independent hilar veins) has important implications for both anatomical interpretation and radiological diagnosis [4]. Given the limited number of reports and variability in terminology, cumulative case estimates should be interpreted cautiously (Figure 2).

**Figure 2:**
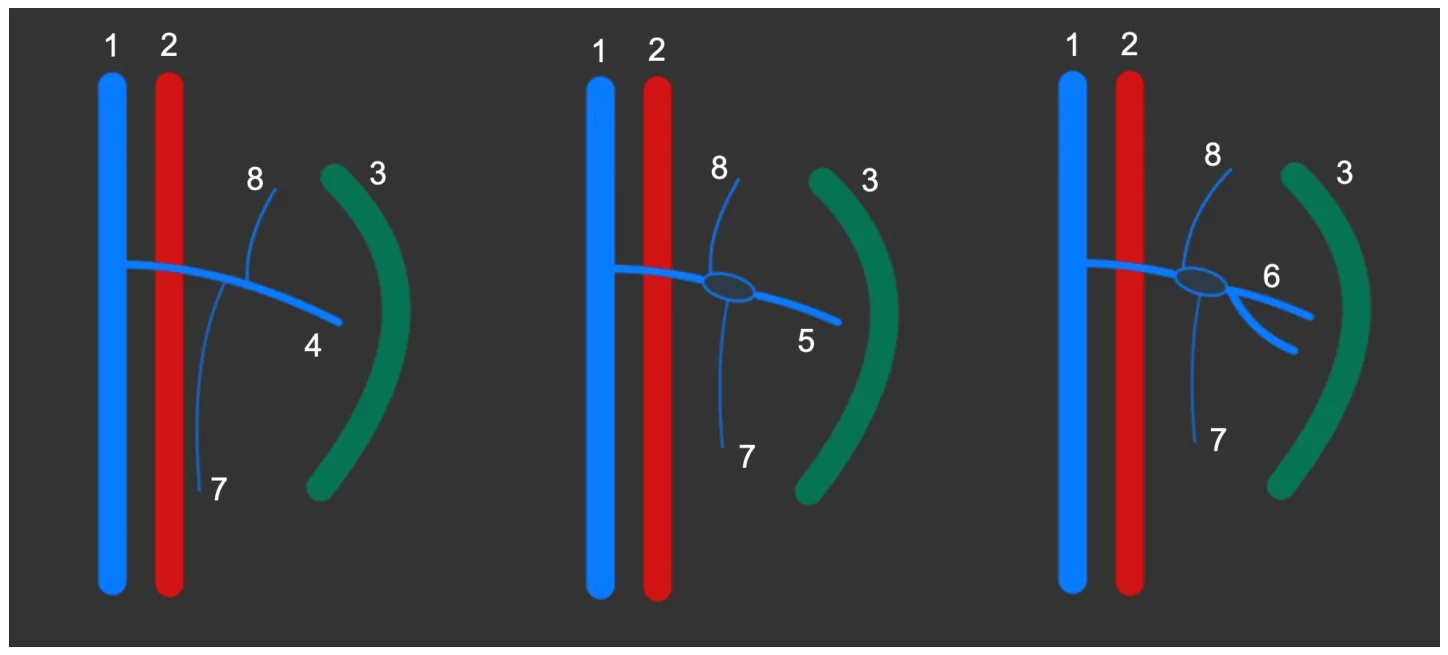
Schematic representation of typical anatomy (**left side**); left renal vein fenestration (**middle**); and left renal vein pseudo-fenestration (**right side**)**.** 1—inferior vena cava; 2—abdominal aorta; 3—kidney hilum; 4—typical left renal vein; 5—fenestration; 6—pseudo-fenestration; 7—left gonadal vein; 8—left suprarenal vein [4]. The present case adds further high-resolution imaging evidence supporting the existence and recognizability of LRV pseudo-fenestration. Radiologically, pseudo-fenestration must be differentiated from a circumaortic LRV, which physically encircles the abdominal aorta, and true LRV duplication, where two independent veins drain directly into the inferior vena cava. Furthermore, it is distinct from late venous confluence, which involves the delayed joining of hilar tributaries without the formation of a characteristic venous window [4]. LRV development occurs between 4 and 8 weeks of gestation through the formation, anastomosis, and selective regression of the subcardinal, supracardinal, and posterior cardinal veins [5]. The ventral (preaortic) and dorsal (retroaortic) components normally fuse into a single preaortic LRV via the inter-subcardinal and sub-supracardinal anastomoses [5]. Anatomical variants arise when this fusion is incomplete, asymmetric, or temporally altered. Incomplete fusion or persistence of both ventral and dorsal limbs at the level of the renal hilum can produce either a true fenestration or the pseudo-fenestration pattern observed here [4]. In the present case, the coexistence of two distinct hilar venous outflows with subsequent convergence is most consistent with delayed or partial coalescence of the sub-supracardinal anastomosis, allowing the inferior hilar vein to persist before joining the dominant venous channel [6,7]. The clinical implications of this variant are substantial. In the context of laparoscopic donor nephrectomy—where the left kidney is generally preferred due to its longer venous pedicle—failure to recognize a pseudo-fenestration preoperatively may lead to misidentification of one limb as the main LRV, with potential consequences including incomplete ligation, inadequate hemostasis, or iatrogenic injury to adjacent structures [8]. This risk is accentuated in minimally invasive approaches, where limited visualization increases reliance on preoperative imaging. Similarly, during venous sampling of the left suprarenal vein, it is the superior limb of the fenestration—or, in pseudo-fenestrations, the dominant superior venous trunk—that receives adrenal drainage, and targeted catheterization must account for this configuration [4]. Interventional procedures such as gonadal vein embolization or splenorenal shunt creation likewise depend on precise delineation of venous anatomy to avoid technical failure or complications. In all these scenarios, CT with multiplanar reconstruction and three-dimensional volume rendering provides the spatial resolution necessary to distinguish between pseudo-fenestration, true fenestration, and duplication of the LRV. Advanced multidetector CT, including three-dimensional and dynamic reconstruction techniques, has been shown to provide high-resolution vascular mapping and improved anatomical delineation, thereby enhancing preoperative planning and reducing procedural risk [2,4,9]. Surgical planning is essential to ensure vascular protection against inadvertent traction or hemorrhage and to mitigate the risk of vascular entrapment by precisely identifying any structures traversing the anomalous venous window [4].

## Data Availability

The original data presented in this study are available on reasonable request from the corresponding author. The data are not publicly available due to privacy concerns.

## Supplementary Materials

The following supporting information can be downloaded at https://www.mdpi.com/article/10.3390/reports9030299/s1. Video S1: Coronal reconstruction of the computed tomography (CT) scan demonstrating the left renal vein pseudo-fenestration.

## Abbreviations

The following abbreviations are used in this manuscript:

AA	Abdominal aorta
CT	Computed tomography
IVC	Inferior vena cava
LGV	Left gonadal vein
LRA	Left renal artery
LRV	Left renal vein
LRV*	Pseudo-fenestrated left renal vein
SRV	Suprarenal vein
